# Population Dynamics of *Anopheles albimanus* (Diptera: Culicidae) at Ipetí-Guna, a Village in a Region Targeted for Malaria Elimination in Panamá

**DOI:** 10.3390/insects9040164

**Published:** 2018-11-16

**Authors:** Lisbeth Amarilis Hurtado, Chystrie A. Rigg, José E. Calzada, Sahir Dutary, Damaris Bernal, Susana Isabel Koo, Luis Fernando Chaves

**Affiliations:** 1 Departamento de Análisis Epidemiológico y Bioestadísticas, Instituto Conmemorativo Gorgas de Estudios de la Salud, Apartado Postal 0816-02593, Panamá, Republic of Panama; lhurtado@gorgas.gob.pa (L.A.H.); sdutary@gorgas.gob.pa (S.D.); 2 Departamento de Investigación en Parasitología, Instituto Conmemorativo Gorgas de Estudios de la Salud, Apartado Postal 0816-02593, Panamá, Republic of Panama; chrigg@gorgas.gob.pa (C.A.R.); jcalzada@gorgas.gob.pa (J.E.C.); 3 Departamento de Investigación en Entomología Médica, Instituto Conmemorativo Gorgas de Estudios de la Salud, Apartado Postal 0816-02593, Panamá, Republic of Panama; dbernal@gorgas.gob.pa (D.B.); skoo@gorgas.gob.pa (S.I.K.); 4 Instituto Costarricense de Investigación y Enseñanza en Nutrición y Salud (INCIENSA), Apartado Postal 4-2250, Tres Ríos, Cartago, Costa Rica; 5 Programa de Investigación en Enfermedades Tropicales (PIET), Escuela de Medicina Veterinaria, Universidad Nacional, Apartado Postal 304-3000, Heredia, Costa Rica

**Keywords:** human landing catch, *Plasmodium*, climate change, metapopulation, Comarca Madungandí

## Abstract

*Anopheles albimanus* Wiedemann is a major malaria vector in Mesoamerica and the Caribbean whose population dynamics, in response to changing environments, has been relatively poorly studied. Here, we present monthly adult and larvae data collected from May 2016 to December 2017 in Ipetí-Guna, a village within an area targeted for malaria elimination in the República de Panamá. During the study period we collected a total of 1678 *Anopheles* spp. mosquitoes (1602 adults and 76 larvae). Over 95% of the collected *Anopheles* spp. mosquitoes were *An. albimanus*. Using time series analysis techniques, we found that population dynamics of larvae and adults were not significantly correlated with each other at any time lag, though correlations were highest at one month lag between larvae and adults and four months lag between adults and larvae. Larvae population dynamics had cycles of three months and were sensitive to changes in temperature with 5 months lag, while adult abundance was correlated with itself (1 month lag) and with the normalized difference vegetation index (NDVI) with three months lag. A key observation from our study is the absence of both larvae and adults of *An. albimanus* between January and April from environments associated with Guna population’s daily activities, which suggests this time window could be the best time to implement elimination campaigns aimed at clearing *Plasmodium* spp. parasites from Guna populations using, for example, mass drug administration.

## 1. Introduction

Panamá is the nation with the highest economic growth in Mesoamerica [1], with a long history of accomplishments [2,3] and landmarks in the study [4,5,6] and control of mosquito vectors of diseases [7] that occurred in Panamá’s territory during the development of the Canal Zone as a colonial possession, for most of the 20th century, of the United States of America [8]. However, Malaria remains an important vector-borne infectious disease in the República de Panamá [9,10]. In Panamá, where an estimate of 900 cases occurred in 2016 [11], malaria disproportionally impacts indigenous populations, where over 90% of the confirmed malaria infections occur [9,12]. The main indigenous group affected by malaria in Panamá are the Gunas [9,13,14], a Chibcha-related ethnic group that has led the struggle for indigenous autonomy within the República de Panamá [15]. In general, indigenous populations living in the “Comarcas” have a low human development when compared with other provinces in Panamá, and most health indicators for indigenous populations lag well behind those observed in the rest of Panamá [16,17].

Since 2000, around half of the malaria cases occurring in Panamá have been concentrated in Comarca Madungandi, especially in the area surrounding Lake Bayano [18], a man-made artificial lake which historically has been associated with outbreaks of several arboviruses [19]. Research on these outbreaks suggested they were associated with changes in mosquito vector populations, triggered by the large scale landscape transformation necessary to create Lake Bayano, which affected common malaria vectors and other mosquito species [19]. Our research has shown that malaria transmission is sensitive to global climatic phenomena, for example, El Niño Southern Oscillation (ENSO) phases have been associated with changes in malaria transmission in Guna Yala [20] and Madungandí [18], the Comarcas where most of the Gunas live in Panamá. Recent research efforts have been made to understand the distribution and diversity of malaria vector species in the República de Panamá, both at the national scale [21], and in endemic regions [22], the population genetics of dominant vector species [23,24] and their insecticide resistance patterns [25,26]. Nevertheless, how weather impacts the population dynamics of major malaria vectors in Panamá remains, for the most, unknown. This is a major knowledge gap, as vulnerability of Guna populations to malaria transmission changes associated with ENSO is likely modulated by changes in mosquito populations dynamics driven by climatic change and weather variability patterns [18,20,27,28]. Moreover, this lack of knowledge is in sharp contrast with the knowledge of African malaria vectors, where clear association patterns between vector population dynamics, weather patterns and malaria transmission have been described [29,30,31,32,33,34,35]. Similarly, studies on the spatial distribution of malaria vectors in Mesoamerica have suggested that vegetation growth and remotely sensed data associated with vegetation growth are useful to predict the abundance of *Anopheles* spp. mosquitoes [36,37,38,39], but no study has looked at the temporal dynamics of association between *Anopheles* spp. mosquitoes and remotely sensed data on vegetation growth in Panamá, and more generally in Mesoamerica. In that sense, our hypothesis is that population dynamics of common *Anopheles* spp. mosquito vectors of malaria parasites might be associated with weather variables and the Normalized Difference Vegetation Index (NDVI), an index estimated from satellite images, which is associated with vegetation growth dynamics [40]. To test that hypothesis, we conducted a 20-month-long study on the anopheline fauna, sampled monthly as both larvae and adults, and evaluated the association of the abundance of these mosquito stages with weather variables and the NDVI for Ipetí-Guna, a village in Lake Bayano’s basin, Comarca Guna de Madungandi, targeted for malaria elimination in the República de Panamá, and without any previous longitudinal study on *Anopheles* spp. population dynamics.

## 2. Materials and Methods 

### 2.1. Study Site

**Figure 1:**
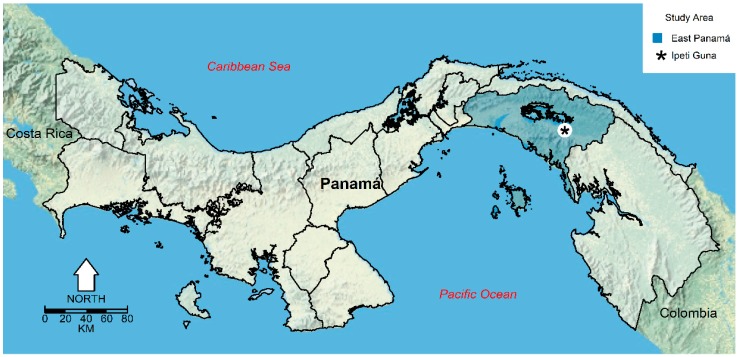
República de Panamá map, highlighting East Panamá province and the location of Ipetí-Guna near the southeastern shore of Lake Bayano. This map was made using as a base a public domain map from the US National Park Service [41].

Our study was done in Ipetí-Guna (8°58′12″ N, 78°30′36″ W) a Guna indigenous village located in Comarca Madungandí, Distrito de Chepo, Panamá Province, República de Panamá (Figure 1). At Ipetí-Guna, over 95% of the houses have a unique room with earthen floors. Construction materials mainly consist of palm fronds used to build walls and thatched roofs. Over 99% of the houses have eaves for ventilation, which are known to favor the infestation by *Anopheles* spp. [4,18]. According to the 2010 National Panamá census [17] Ipetí-Guna has a total population of 711 people in 83 households. Overall, this village has a low coverage of basic services and most of the inhabitants live in conditions of poverty, for example, over 98% of the people access water from the river, 100% of the household do not have a sanitation system and over 80% of the households (with an average size of 5 members) have a monthly income below 250 US $ per month, i.e., well below the poverty line of 3 US $ per person per day [17]. Moreover, less than 1% of the population is covered by Panamá’s social security [17].

### 2.2. Mosquito Sampling

**Figure 2:**
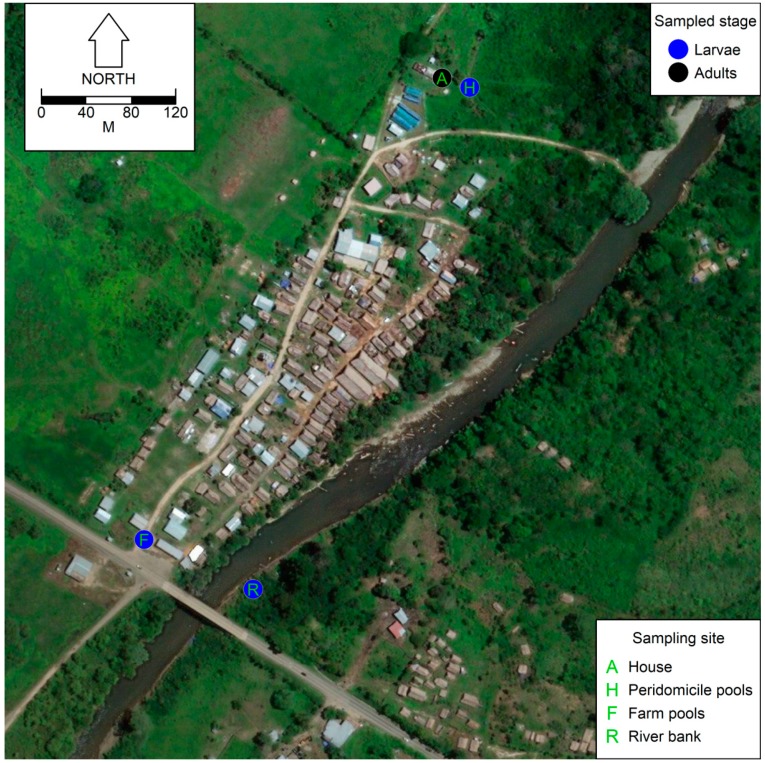
Map of Ipetí-Guna, showing the four sampling locations. Mosquito stages sampled are highlighted by colors, and sampling location codes are indicated by letters. For further details please refer to the letters and colors indicated in the inset legends. This map was made using as a base an image from Google Earth [46].

Mosquitoes, adults and larvae, were monthly collected from May 2016 to December 2017. *Anopheles* spp. adults mosquitoes were collected by human landing catch [22], a highly sensitive mosquito sampling method for anopheline mosquitoes [42], at the peridomicile of a housing cluster in Ipetí-Guna (Figure 2). This housing cluster was chosen given the abundance of nearby positive larval habitats. It is worth highlighting that distance from the sampling location to the huts in the housing cluster ranged between 5 m and 10 m. The adult mosquito collections were made over three consecutive nights between 18:00 h and 21:00 h by two trained collectors that switched positions to minimize bias in the abundance estimates [41]. To minimize the infection risk, catches followed World Health Organization (WHO) biosecurity guidelines [43]. *Anopheles* spp. larvae were collected by dipping larval habitats near the households, but also on the Ipetí-Guna River banks and in the farmlands of the community (Figure 2), following the procedure recommended by WHO, of ten dips per square m at a sampling location [43]. Larvae and adults mosquitoes were placed in Petri dishes, which were coded by collection site and date, and then mosquitoes were morphologically identified using a dissection scope and taxonomic keys for the anophelines of Mesoamerica [44,45] and the reference collection at the Instituto Conmemorativo Gorgas de Estudios de la Salud (ICGES). 

### 2.3. Weather Data

Temperature and rainfall data were obtained from gridded databases. The data were extracted from the Royal Netherlands Meteorological Institute website, using the KNMI explorer [47]. The specific gridded databases that we employed were: (i) NOAA CPC Morphing Technique (“CMORPH”) database to obtain monthly rainfall data, which has a resolution of 0.25° [48]; (ii) NOAA Global Historical Climatology Network version 2 and the Climate Anomaly Monitoring System (GHCN_CAMS 2m model) for temperature, which has a spatial resolution of 0.5° [49]. To create a seasonal climate profile, with monthly boxplots for the 12 months of the year, monthly data were downloaded from 1998 to 2017 for the study area. 

### 2.4. Vegetation Data

We employed images for the NDVI from the biweekly, 250 m resolution, vegetation product (M*D13A3), courtesy of the NASA Land Processes Distributed Active Archive Center, USGS/Earth Resources Observation and Science (EROS) Center, Sioux Falls, South Dakota [50]. We used the R package MODIStsp [51] to download and clip NDVI images from the study period. Further GIS procedures for the downloaded images were made using the package raster also in the statistical software R [52]. Briefly, all images from the study period were stacked into a geotiff, from which the monthly values were extracted, generating a time series in the process [20]. 

### 2.5. Time Series Analysis

To study the abundance patterns of *Anopheles albimanus* adults and larvae we followed a standard procedure for time series analysis, where we first inspected the autocorrelation patterns of both time series. We started by inspecting the autocorrelation function (ACF) and the partial autocorrelation function (PACF). Briefly, the ACF depicts the correlation of the time series with itself through time, while the PACF does so considering the correlation between consecutive time lags [53]. With information from both of these functions it was possible to identify time lags associated with population size, of both larvae and adults, at any given time. This information was then used to fit a null model that was used to pre-whiten time series from all the environmental covariates described before. Pre-whitening is a procedure that removes any potential similar autocorrelation from the environmental covariates time series, thus ensuring cross-correlation functions (CCF) show associations that do not emerge from time series having similar autocorrelation structure. To estimate CCFs, we used residuals from the null models fitted to the adults and larvae data and the pre-whitened time series from each environmental covariate. Based on the resulting CCFs we chose lags to fit time series models including the covariates that were associated with mosquito abundance by stage (adults and larvae). The time series models we fitted can be described by the following equation:

(1) $$\;y_{t}=\mu +\underset{x}{\sum }\varphi_{x}\left(y_{t-x}-\mu \right)+\underset{x}{\sum }\alpha_{x}\left(cov_{t-x}-mean\left(cov\right)\right)+\varepsilon_{t}\;$$

where $y_{t}$ is the mosquito abundance at time *t, x* indicates the time lag, μ is the average mosquito abundance during the study period, $\varphi_{x}$ the autoregressive coefficients, *cov* indicates a covariate (weather and vegetation variables described above), $\alpha_{x}$ the coefficients for the covariates and $\varepsilon_{t}$ is the error, which was assumed to be normal, identical and independent [53]. To select the environmental covariates, we used backward elimination guided by the minimization of the Akaike Information Criterion (AIC). AIC is a parameter that trades-off model goodness-of-fit and parameter number, and whose minimization can be used to select among models with a similar number of parameters [53]. For the best model, error assumptions were verified using standard procedures for time series analysis [53]. 

## 3. Results

During the study period we collected a total of 1678 mosquitoes, 1602 were adults and 76 larvae. Over 95% of the collected *Anopheles* spp. mosquitoes were *An. albimanus*, 1585 adults and 71 larvae (Table 1). The monthly abundance (±S.D.) of *An. albimanus* was 79.25 ± 96.40 for adults and 3.55 ± 4.70 for larvae. Other anopheline mosquito species collected at the study site included: *Anopheles punctimacula* Dyar & Knab, and *Anopheles punctipennis* (Say), both of these species collected as adults and larvae, and *Anopheles apicimacula* Dyar and Knab, collected only as adult (Table 1). 

**Figure 3:**
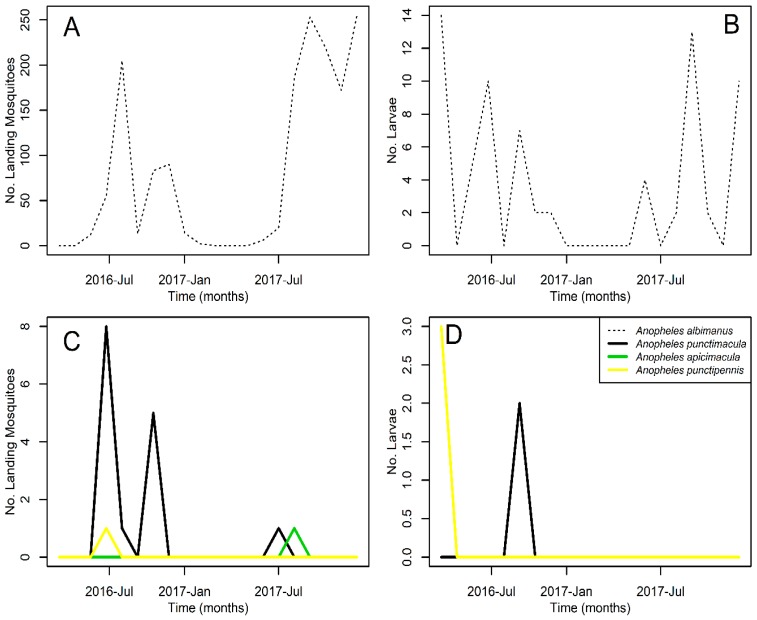
Monthly mosquito abundance time series (**A**) *Anopheles albimanus* adults (**B**) *Anopheles albimanus* larvae (**C**) Less common *Anopheles* spp. adults (**D**) Less common *Anopheles* spp. larvae. Adults were sampled by human landing catch, and larvae using a dipper. Collected *Anopheles* spp. are presented in the inset legend of panel (**D**). All time series correspond to the studied period, from May 2016 to December 2017. For further details about the sampling, please refer to the mosquito sampling section in the methods.

The mosquito *An. albimanus* was not only the most abundant anopheline mosquito species, but also the most persistent, being present at the study site 75% and 60% of the times mosquitoes were sampled, respectively, as adults and larvae. *An. albimanus* was absent in adult mosquito samples from April to May of 2016 and from February to April of 2017 (Figure 3A), while larvae were absent in May and August 2016, and from January to May, and July of 2017 (Figure 3B). Meanwhile, all other *Anopheles* spp., had very low persistence (Table 1) and were caught mainly during 2016 as both adults (Figure 3C) and larvae (Figure 3D).

**Figure 4:**
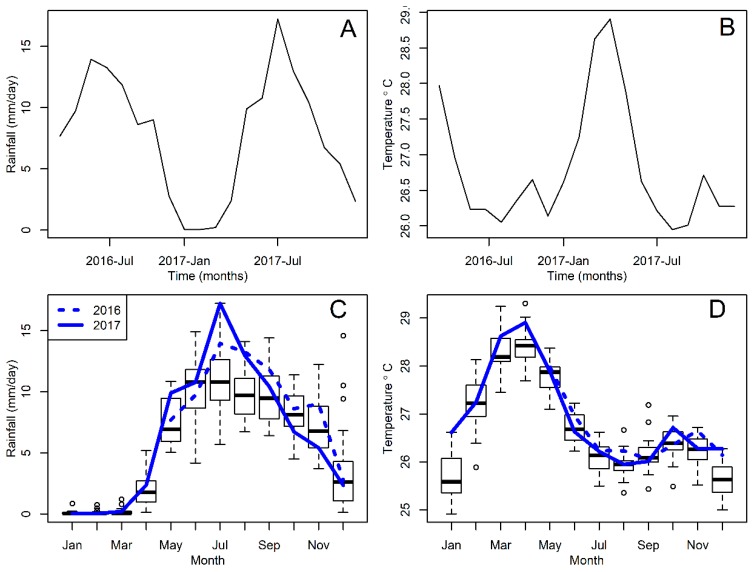
Monthly weather time series (**A**) Rainfall (**B**) Temperature (**C**) Seasonal rainfall (**D**) Seasonal temperature. In panels (**A**,**B**) the time series correspond to the studied period, from May 2016 to December 2017. In panels (**C**,**D**) each monthly boxplot represents the distribution of records between 1998 and 2017, and the studied period is highlighted by blue lines described in the inset legend of panel (**C**). In the boxplots, the line at the center of each box indicates the median of the collected weather records.

The persistence and abundance patterns of *An. albimanus* seemed to track changes in rainfall (Figure 4A) and temperature (Figure 4B) at the study site, with both adult and larvae being absent during the dry season that spans from January to April (Figure 4C) and during the hottest season, with the highest temperatures, from February to May (Figure 4D). It is worth highlighting that both rainfall and temperature had record high values during the study period, where rainfall in July 2017, at the peak of the rainy season (Figure 4C), was well above the median, and temperatures were hotter than usual in January, April and December (Figure 4D) when compared with the distribution of records from 1998 to 2017.

**Figure 5:**
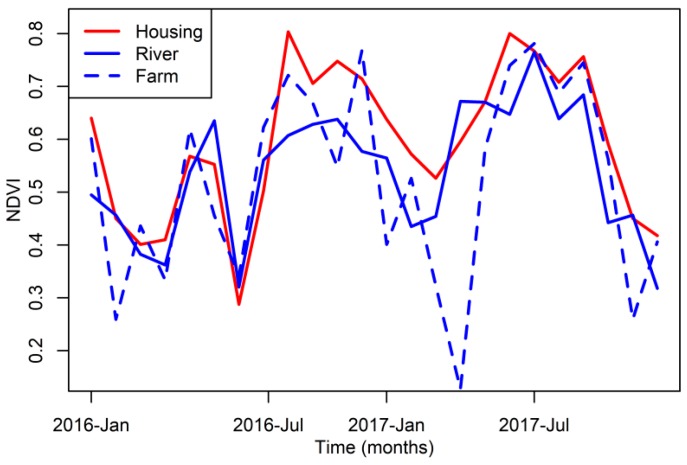
Monthly NDVI records. For line codes please refer to the inset legend. House pools and the adult sampling location were in the same 250 m MODIS pixel, referred as Housing in the inset legend.

**Figure 6:**
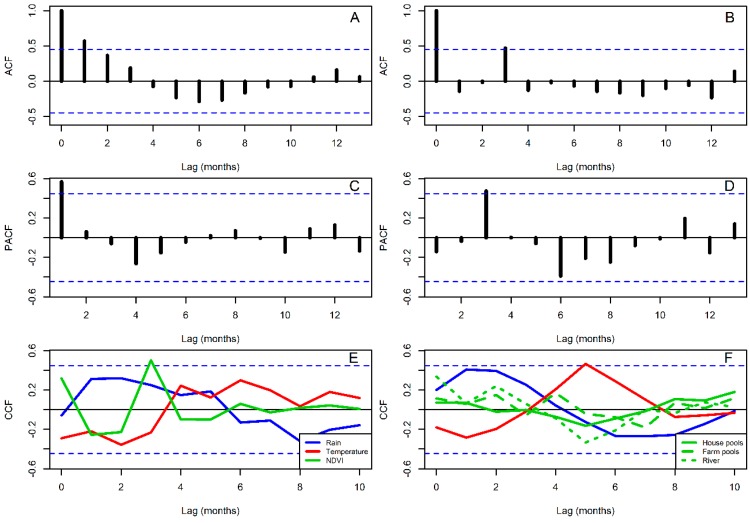
Correlation functions (**A**) *Anopheles albimanus* adult abundance autocorrelation function, ACF; (**B**) *An. albimanus* larvae abundance ACF; (**C**) *An. albimanus* adult abundance partial ACF, PACF; (**D**) *An. albimanus* larvae abundance PACF. Cross correlation functions, CCF, between *An. Albimanus*; (**E**) adults and rainfall, temperature and NDVI, based on the data for House (**F**) larvae and rainfall, temperature and NDVI, based on the data for house pools, farm pools and the river. The inset legend of panel (**E**) shows the color coding for the different environmental variables, while the inset legend of panel (**F**) shows the line coding for the NDVI measured at the different sampling locations. In each plot, blue dashed lines indicate the confidence intervals within which correlations are expected to arise by random (*p* > 0.05).

Figure 5 shows the NDVI of the sampling sites for 2016 and 2017, showing the Housing pixel, where larvae and adults were both sampled (Figure 2), had the highest NDVI during the entire study period, followed by the river site and the farm site, which reached low values during the dry season. Autocorrelation functions from the *An. albimanus* abundance time series suggest that adults and larvae had different patterns of temporal abundance association, where adults had a decaying autocorrelation function (Figure 6A), but larvae had a cyclic autocorrelation function (Figure 6B). Both of these were patterns confirmed by the partial autocorrelation functions, which suggested a first order autoregressive process for the adult abundance, i.e., the time series was significantly correlated (*p* < 0.05) up to one month lag (Figure 6C), while, by contrast, larvae abundance was significantly associated (*p* < 0.05) in a pattern that suggested cycles with a three month lag (Figure 6D). Regarding the environmental covariates, adults were significantly correlated (*p* < 0.05) with NDVI with a three month lag (Figure 6E), while larvae were associated (*p* < 0.05) with temperature with a five month lag (Figure 6F).

**Figure 7:**
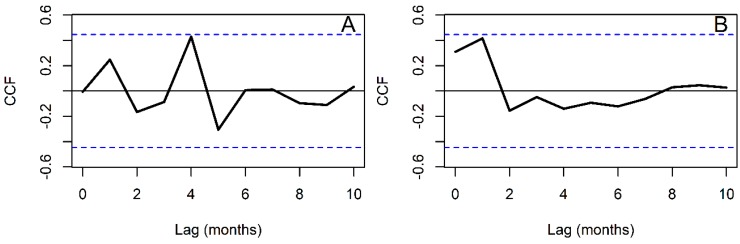
Cross correlation functions between *Anopheles albimanus* stage abundance (**A**) Adults as a function of larvae (**B**) Larvae as a function of adults. In each plot, blue dashed lines indicate the confidence intervals within which correlations are expected to emerge by random (*p* > 0.05).

Meanwhile, the association between adults and larvae was not statistically significant at any time lag, yet it had a peak at four month lag (Figure 7A). The association between larvae and adults was highest at one month lag (Figure 7B), but not statistically significant, yet higher than the four month lag association between adults and larvae. 

The significant lags found in the different cross correlation functions were used to fit time series models described in the following lines. Table 2 shows the results of the model selection process for the time series model explaining *An. albimanus* adult abundance. As shown in Table 2, the autoregressive model including NDVI with a three month lag outperformed an autoregressive model. 

Parameter estimates for the best *An. albimanus* adult abundance model are presented in Table 3, showing that *An. albimanus* adult abundance was positively associated with NDVI (*p* < 0.05), indicating that mosquito abundance increases following vegetation growth at the study site. 

Table 4 shows the results of model selection for variables associated with *An. albimanus* larvae abundance. As shown in Table 4, the best model included temperature with five month lag as a covariate, adult abundance with one month lag and a seasonal autoregressive component with three month period. Although the associations between *An. albimanus* stages, i.e., adult and larvae, were not statistically significant, we considered the one month lag adult abundance in the *An. albimanus* larvae model because the correlation was slightly larger than that observed between adults and larvae. 

Parameter estimates for the best model describing the population dynamics of *An. albimanus* larvae are presented in Table 5, which shows a positive and significant (*p* < 0.05) association between larvae abundance and temperature with 5 months lag, and a positive but non-significant association with *An. albimanus* adult abundance with 1 month lag.

## 4. Discussion

Although malaria has been significantly decreasing in Mesoamerica [54], this vector-borne disease remains a public health problem in the region, particularly in Panamá, the only country in Mesoamerica that did not reach the millennium development goal of a 75% reduction of malaria transmission [55]. In Panamá, it has become increasingly clear that malaria mainly affects the Gunas [9,22]. The vulnerability of this group to malaria is such that transmission of highly pathogenic malaria parasites, often resistant to antimalarials [13,14], has been clonal, indicative of epidemic transmission as revealed by low parasitic genetic diversity, and geographically widespread [56]. Malaria transmission patterns in Panamá, although without a clear seasonality, have interannual transmission cycles linked to ENSO [18,20], a global climatic phenomenon associated with changes in malaria transmission worldwide [57,58,59,60,61]. This situation calls for increased efforts to define and implement focalized activities to control and eliminate malaria from the Gunas, where their overarching situation of social exclusion, poverty, and different cultural beliefs [9,22] on top of their poor housing quality [12,22], exclude a fast-paced change favoring improved living conditions. For example, housing improvement [62], a change that has been decisive for malaria disappearance from diverse settings around the world [8,63,64,65,66,67,68,69,70,71] is out of reach for Guna communities in Panamá. In this context, insights from studying malaria mosquito vector ecology could be useful to guide malaria transmission reduction [72,73]. 

Our longitudinal study revealed a strong seasonality in *An. albimanus*; its abundance greatly decreases during the dry season, January to April, a pattern observed for anophelines elsewhere in the New World [74,75,76,77,78]. This reduced mosquito abundance pattern makes the dry season ideal for interventions aimed at eliminating parasites from the human population, for example, via mass drug administration (MDA) [79,80,81]. MDA can be adapted to reduce malaria transmission among indigenous populations with unique cultural characteristics [82], like the Gunas. Indeed, models of adaptive management in changing environments have already proved successful for natural resource extraction and food sovereignty among the Gunas [83]. Timing parasite clearance interventions from Guna populations during the dry season is expected to be optimal, mainly because the likelihood of any transmission, as parasites are cleared from the human population is greatly reduced when mosquitoes are scarce [76]. Moreover, MDA has been successfully applied for malaria elimination from indigenous populations elsewhere [80,84]. 

Our results confirm the importance that vegetation growth changes have on regulating the abundance of host seeking adult mosquitoes [42,74], given the time series model for adults indicated that peaks in mosquito abundance follow NDVI changes, with a lag of three months. The three month lag could reflect at least a couple of phenomena, delayed density dependence and resonant generational cycles. In delayed density dependence, population growth in the larvae, which are sensitive to vegetation growth changes [36,37,38], is seen with a delay in the adults [85]. Meanwhile, in resonant generational cycles [86,87,88], environmental impacts on population size have a build-up time to be observed in the adult mosquito population and can be enhanced by a positive autocorrelation structure in the environment, something that could also explain the 5 month lag for the impact of temperature in aquatic mosquito, i.e., larvae, population growth, as has been observed in other mosquito vectors of pathogens [89]. These relationships with environmental factors are also important to design interventions aimed at reducing the contact between the Gunas and malaria vector populations, since, for example, in Sub-Saharan Africa it is known that adherence to use insecticide treated nets is influenced by perceived mosquito nuisance [90] and the concomitant perceived malaria transmission risk [91]. In that sense, the best timing for interventions aimed at reducing contact between humans and vectors, or suppressing vector populations, will be during the wet season, for example, three months after peaks in NDVI, which are correlated with peaks in host-seeking mosquitoes, as shown in this study. Among the tools used to reduce the contact between Gunas and mosquitoes, it is unlikely that insecticide treated nets, a tool with an extraordinary success in reducing malaria transmission in Sub-Saharan Africa [92,93], will be effective among the Gunas, as they sleep in hammocks. However, the use of insecticide-treated hammocks [94,95] might be an option that can be adopted by the Gunas as it fits their cultural practices [82].

We think the entomological risk of malaria transmission posed by the three other collected species needs to be further evaluated because all the *Anopheles* spp. we collected have been found infected with malaria parasites [6,96,97,98], and might surge in case strategies aimed at suppressing *An. albimanus* vector populations are implemented in Guna communities. This phenomenon has been commonly observed when knocking down the density of dominant vector species elsewhere [27,28,99].

Finally, some limitations of our study include the limited sampling which was constrained by the lack of economic resources to sample mosquitoes across the sparse Guna villages in Lake Bayano basin, that are extremely difficult to access during the rainy season. Our study was also limited by our ability to find larval habitats at the study site which, although productive, seemed to be very scarce compared with descriptions for African [100,101] and South American [75,78] settings. Although it has been suggested that human landing catch is the best method to sample adult mosquitoes in the Neotropics [42], we think our study could have benefited by using other sampling methods for the adult populations, such as Mosquito Magnet^®^ traps [102]. Also, our environmental data could have been more descriptive of the local environmental conditions of our sampling sites if our budget had allowed us to use data loggers measuring local weather variables [103] instead of resorting to spatially coarsely grained records from gridded databases. We also think that a more frequent sampling, for example, biweekly instead of monthly, could have been helpful to straightforwardly link patterns of larvae and adult abundance, which at the monthly time scale, were not significantly correlated with each other. 

## 5. Conclusions

Our study describes basic features of *An. albimanus* population dynamics, a dominant malaria vector in Mesoamerica [6,21], highlighting *An. albimanus* abundance association with environmental variables at a site where this disease has a large burden on indigenous Guna populations [18]. Based on our observations, we can suggest the use of measures aimed at clearing parasites from the Gunas, for example, mass drug administration, during the dry season from January to April, when host-seeking malaria mosquito abundance is low and the likelihood of transmission is reduced. In contrast, the deployment of interventions aimed at reducing human and vector contact might be more successful during the wet season, when mosquito nuisance might promote the adherence to use protective measures against mosquito bites or lead to perceptible changes in nuisance, when using tools aimed at reducing vector populations, producing a positive feedback on their use and acceptance [104,105]. Fine tuning for the deployment time of interventions affecting malaria vectors can benefit from remotely sensed data of vegetation growth, for example, using NDVI, and also with the use of temperature records, as inferred from the lagged association between these variables and *An. albimanus* abundance, respectively, as adults and larvae. Given our previous findings about the association of ENSO and malaria transmission at Comarca Madungandi [18] and Comarca Guna Yala [20], where ENSO, respectively, has a positive and negative impact on malaria transmission, we think studies on malaria vector population ecology are a priority in Guna Yala, since local weather conditions might have different impacts on *Anopheles* spp. abundance that explain the contrasting impacts of ENSO on malaria transmission. We think that areas like Cartí in Comarca Guna Yala, a town with malaria transmission [20,22] and expected to receive an influx of Gunas from Caribbean islands that are disappearing as result of climate change [106], and for which no malaria related environmental health assessment has been made, are the priority for developing studies designed to understand malaria vector ecology, in order to propose interventions that ensure malaria transmission reduction and elimination from the Gunas in the República de Panamá.

## Figures and Tables

**Table 1:** Monthly mean abundance of *Anopheles* spp mosquitoes, by stage, collected between May 2016 and December 2017 in Ipetí-Guna, Comarca Madungandí, República de Panamá. The column “Total” indicates the total number of individuals collected for each species during the study period, and the column “Persistence” the proportion of the sampling sessions when a given mosquito species was found among the samples.

		Adults			Larvae	
Species	Mean ± SD	Total	Persistence (%)	Mean ± SD	Total	Persistence (%)
*Anopheles albimanus*	79.25 ± 96.40	1585	75	3.55 ± 4.70	71	60
*Anopheles punctimacula*	0.75 ± 2.05	15	15	0.10 ± 0.45	2	5
*Anopheles apicimacula*	0.05 ± 0.22	1	5	—	—	—
*Anopheles punctipennis*	0.05 ± 0.22	1	5	0.15 ± 0.67	3	5

**Table 2:** Model selection for variables explaining *Anopheles albimanus* adult abundance at Ipetí-Guna, República de Panamá. AIC indicates the Akaike Information Criterion for the model, and Variables the covariates considered in each model. In the variables, AR indicates Autoregressive. The best model, with the lowest AIC, is in bold font.

Variables [Lag in Months]	AIC
AR[1]	234.51
AR[1], NDVI[3]	**227.60**

**Table 3:** Parameter estimates for the best time series model explaining *Anopheles albimanus* adult abundance at Ipetí-Guna, República de Panamá. In parameters AR stands for Autoregressive.

Parameter [Lag in Months]	Estimate ± S.D.
Intercept ($\hat{\mu }$)	83.60 ± 39.79 *
AR[1]$\;(\hat{\phi_{1}}$)	0.71 ± 0.16 *
NDVI[3] $\left(\hat{\alpha_{3}^{NDVI}}\right)$	3.85 ± 1.15 *
$\mathrm{Error}\;\mathrm{Variance}\;(\hat{\sigma^{2}}$)	3320

* Statistically significant (*p* < 0.05).

**Table 4:** Model selection for variables explaining *Anopheles albimanus* larvae abundance at Ipetí-Guna, República de Panamá. AIC indicates the Akaike Information Criterion for the model, and Variables the covariates considered in each model. In the variables, SAR indicates Seasonal Autoregressive. The best model, with the lowest AIC, is in bold font.

Variables [Lag in Months]	AIC
SAR[3]	108.60
SAR[3], Adults[1]	107.40
SAR[3], Temperature[5]	102.20
SAR[3], Temperature[5], Adults[1]	**101.87**

**Table 5:** Parameter estimates for the best time series model explaining *Anopheles albimanus* larvae abundance at Ipetí-Guna, República de Panamá. In parameters, SAR stands for Seasonal Autoregressive.

Parameter [Lag in Months]	Estimate ± S.D.
Intercept ($\hat{\mu }$)	2.59 ± 1.53 *
SAR[3]$\;(\hat{\phi_{3}}$)	0.67 ± 0.19 *
Temperature[5] $\left(\hat{\alpha_{5}^{TEMP}}\right)$	1.35 ± 0.44 *
Adults[1] $\left(\hat{\alpha_{1}^{Adults}}\right)$	0.010 ± 0.006
$\mathrm{Error}\;\mathrm{Variance}\;(\hat{\sigma^{2}}$)	6.69

* Statistically significant (*p* < 0.05).
